# Modeling non-hereditary mechanisms of Alzheimer disease during apoptosis in yeast

**DOI:** 10.15698/mic2015.04.199

**Published:** 2015-03-20

**Authors:** Ralf J. Braun, Cornelia Sommer, Christine Leibiger, Romina J. Gentier, Verónica I. Dumit, Katrin Paduch, Tobias Eisenberg, Lukas Habernig, Gert Trausinger, Christoph Magnes, Thomas Pieber, Frank Sinner, Jörn Dengjel, Fred W. v. Leeuwen, Guido Kroemer, Frank Madeo

**Affiliations:** 1 Institute of Cell Biology, University of Bayreuth, 95440 Bayreuth, Germany.; 2 Institute of Molecular Biosciences, NAWI Graz, University of Graz, 8010 Graz, Austria.; 3 BioTechMed-Graz, 8010 Graz, Austria.; 4 Department of Neuroscience, Faculty of Health, Medicine and Life Sciences, Maastricht University, 6229 ER Maastricht, The Netherlands.; 5 FRIAS Freiburg Institute for Advanced Studies, Department of Dermatology, Medical Center, ZBSA Center for Biological Systems Analysis, BIOSS Centre for Biological Signalling Studies, University of Freiburg, 79104 Freiburg, Germany.; 6 HEALTH Institute for Biomedicine and Health Sciences, Joanneum Research, 8010 Graz, Austria.; 7 Division of Endocrinology and Metabolism, Medical University of Graz, 8036 Graz, Austria.; 8 Apoptosis, Cancer & Immunity Laboratory, Team 11, Equipe labellisée Ligue contre le Cancer, INSERM Cordeliers Research Cancer, 75006 Paris, France.; 9 Cell Biology & Metabolomics Platforms, Gustave Roussy Comprehensive Cancer Center, 94805 Villejuif, France.; 10 Pôle de Biologie, Hôpital Européen Georges Pompidou, AP-HP, 75015 Paris, France.; 11 Université Paris Descartes, Sorbonne Paris Cité, 75005 Paris, France.

**Keywords:** microbes, microbial research, unicellular organism, microbiome, cell death, apoptosis, autophagy, aging, neurodegeneration

## Abstract

Impaired protein degradation and mitochondrial dysfunction are believed to contribute to neurodegenerative disorders, including Alzheimer disease (AD). In patients suffering from non-hereditary AD, UBB^+1^, the frameshift variant of ubiquitin B, accumulated in neurons affected by neurofibrillary tangles, which is a pathological hallmark. We established a yeast model expressing high levels of UBB^+1^, and could demonstrate that UBB^+1^ interfered with both the ubiquitin-proteasome system (UPS) and mitochondrial function. More precisely, UBB^+1^ promoted the mitochondrion-localized production of the basic amino acids arginine, ornithine, and lysine, which we identified as the decisive toxic event culminating in apoptosis. Inducing the UPS activity at mitochondria prevented the lethal basic amino acid accumulation and avoided UBB^+1^-triggered cell loss. The arginine/ornithine metabolism is altered in brains of AD patients, and VMS1, the mitochondrion-specific UPS component, co-existed with UBB^+1^ in neurofibrillary tangles. Therefore, our data suggest that aberrant basic amino acid synthesis is a crucial link between UPS dysfunction and mitochondrial damage during AD progression.

We expressed UBB^+1^ in yeast, which recapitulated hallmarks of UBB^+1^ in neurons. Due to its altered C-terminus, UBB^+1^ was a loss-of-function variant of ubiquitin B, which was ubiquitylated by ubiquitin ligases and truncated by the ubiquitin hydrolase Yuh1. Like in neurons, UBB^+1^ accumulation impaired the UPS as indicated by the accumulation of polyubiquitylated proteins, and the delayed turnover of the UPS model substrate ubiquitin-G76V-GFP.

As in neurons, protracted expression of UBB^+1^ and/or the application of stress was needed to induce loss of cell survival, and oxidative stress, which culminated in apoptosis and necrosis. Different UBB^+1^ species turned out to show different levels of cytotoxicity: ubiquitylated UBB^+1^ was found to be more cytotoxic as compared to its non-ubiquitylated variant, and truncation of UBB^+1^ was proposed to be a potential protective mechanism.

Deletion of the major ubiquitin gene *UBI4* increased UBB^+1^ lethality. In contrast, promoting the UPS capacity of yeast cells by expressing or stabilizing the major UPS transcriptional activator Rpn4 relieved UBB^+1^-triggered cytotoxicity, but not in the absence of *UBI4*. Thus, the UPS capacity and the ratio of mutant to wild-type ubiquitin, with UBB^+1^ as a potential competitive inhibitor of wild-type ubiquitin, dictated the cytotoxicity of UBB^+1^ in yeast.

Mitochondria were pivotally involved in executing UBB^+1^-triggered cell death. Mitochondrial and oxidative stress coincided in yeast cells expressing UBB^+1^. Increased cellular oxygen consumption and elevated mitochondrial membrane potential co-occurred with the depletion of Rip1, an essential component of the respiratory chain, and with a dramatic loss of cellular ATP. These data hint at hyperactive mitochondria and a metabolic crisis in yeast cells expressing UBB^+1^.

A quantitative proteomic approach (SILAC) identified the accumulation of the enzymes Arg5,6, Arg8, and Lys1 involved in the production of arginine, ornithine, and lysine, in mitochondrial extracts from cells expressing UBB^+1^. Consistently, using a metabolomics approach, we observed the cellular accumulation of these basic amino acids in cells with high levels of UBB^+1^, suggesting the accumulation of functionally active enzymes in the mitochondrial fraction. Depletion of these enzymes relieved UBB^+1^-triggered cell death, pointing to a decisive role of aberrantly increased basic amino acid production at mitochondria in the execution of UBB^+1^ lethality.

UBB^+1^-triggered mitochondrial stress and aberrantly increased basic amino acid synthesis were prevented by the stimulation of the UPS activity at mitochondria. More specifically, high levels of the mitochondrial UPS component Vms1 reduced the amount of the enzymes Arg5,6, Arg8, and Lys1 in mitochondrial extracts upon UBB^+1^ expression, and reduced the levels of the basic amino acids arginine, ornithine, and lysine. Consistently, mitochondrial function and bioenergetics were recovered. Since high levels of Vms1 did not alter the steady-state-level of UBB^+1^, these data propose that Vms1 interrupted the lethal signaling cascade triggered by UBB^+1^ at the level of mitochondria.

Human VMS1 co-existed with UBB^+1^ and mitochondrial VDAC1 in tau-containing neurofibrillary tangles in hippocampal neurons of AD patients and aged non-demented controls with tau pathology. Based on these data, we propose that VMS1-dependent mitochondrial proteostasis might retard the neuronal dysfunction triggered by the accumulation of aberrant tau and UBB^+1^ (Figure 1).

**Figure 1:**
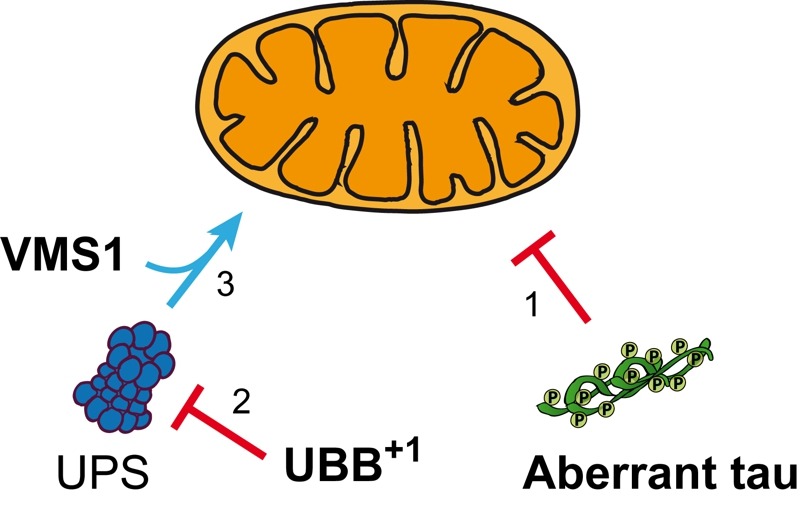
FIGURE 1: Hypothetical model for preventing AD-associated mitochondrial dysfunction triggered by the accumulation of UBB^+1^ and aberrant tau. Hyperphosphorylated forms of the microtubule-associated protein tau interfere with mitochondrial function (1). In parallel, the cellular accumulation of UBB^+1^ induces UPS dysfunction (2) thereby indirectly impairing mitochondria. The mitochondrion-associated UPS, which depends on VMS1, ensures the local protein quality at mitochondria (3) and by this way protects from neuronal cell loss elicited by damaged mitochondria.

Recent studies from several groups demonstrated that UPS dysfunction can lead to mitochondrial dysfunction and vice versa, and altered arginine/ornithine metabolism has been described in both aged human brains and brains from AD patients. Our data demonstrated that high levels of UBB^+1^ interfered with both the UPS and mitochondria. We further showed that the basic amino acid synthesis at mitochondria was induced by UPS dysfunction due to UBB^+1^ accumulation. Future studies should address the following questions:

- By which cellular mechanisms does UPS dysfunction lead to the increased basic amino acid synthesis at mitochondria? Are the observed increased steady-state levels of functional enzymes in the mitochondrial matrix caused by increased cytosolic protein synthesis, and/or by increased mitochondrial import of these enzymes, and/or by decreased enzyme degradation in the matrix? How does UPS dysfunction influence these cellular processes, and is this specific for UBB^+1^-induced UPS impairment?

- How does the cellular accumulation of basic amino acids trigger the increased mitochondrial dysfunction and cytotoxicity? Is the lethal effect exerted directly on mitochondria? Or are other organelles involved, such as the vacuole/lysosome?

The answers to these issues could potentially reveal the functional link between two major hallmarks of AD, namely the UPS and mitochondrial dysfunctions, and thus open an avenue for further research.

